# Supplementary material for: Pioneer and repressive functions of p63 during zebrafish embryonic ectoderm specification
Source: Nat Commun. 2019 Jul 11;10:3049. doi: 10.1038/s41467-019-11121-z (PMC6624255; doi:10.1038/s41467-019-11121-z)
Supplement: Supplementary file 1 — Supplementary Information [file 41467_2019_11121_MOESM1_ESM.pdf]

Supplementary Information

Pioneer and repressive functions of p63 during zebrafish embryonic ectoderm specification

Santos-Pereira et al.

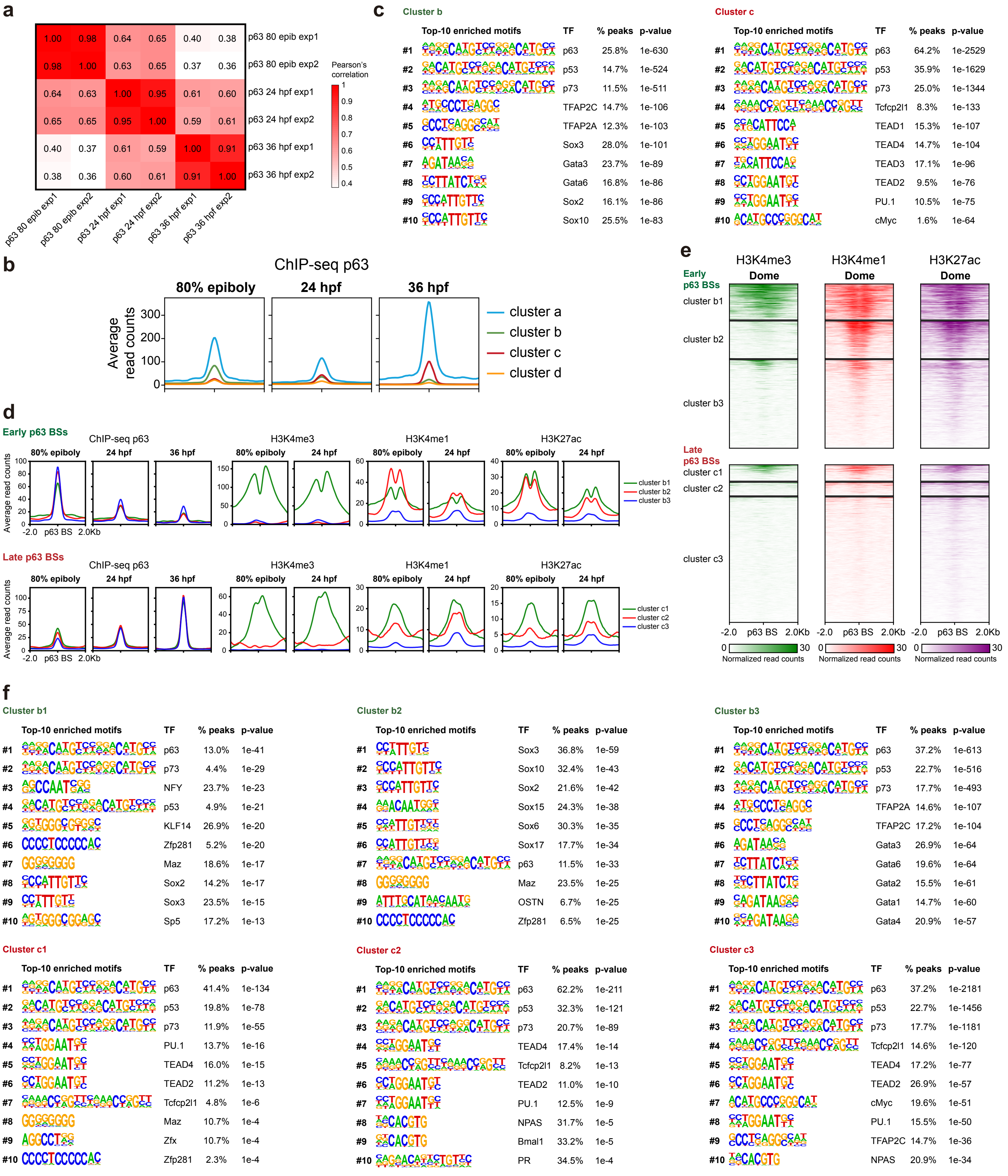

Supplementary Figure 1

Epigenomic and motif analyses of p63 binding sites (BSs) during embryo development.

a, Heatmap showing Pearson's correlations between p63 ChIP-seq experiments in different stages. **b,** Average profile of the 30,597 p63 binding sites (BSs) obtained for 80% of epiboly, 24 hpf and 36 hpf stages clustered in 4 groups by *k*-means clustering (see Figure 2b). **c,** Motif enrichment analysis of the early (cluster b) and late (cluster c) p63 binding sites (BSs) from Figure 2b. The top-10 motifs are represented with their position in the top-10, motif logos, transcription factor (TF) names, percentage of peaks with the motif and enrichment p-value. **d,** Average profiles of early (top) and late (bottom) p63 BSs in 80% of epiboly, 24 hpf and 36 hpf, clustered according to chromatin marks H3K4me3, H3K4me1 and H3K27ac in 80% of epiboly and 24 hpf (see Figure 2e). **e,** Heatmaps of H3K4me3, H3K4me1 and H3K27ac signal in dome stage for the clustered early (top) and late (bottom) p63 BSs (see Figure 2e). **f,** Motif enrichment analysis of the clusters of p63 BSs from Figure 2e. OSTN, Oct4-Sox2-Tcf-Nanog.

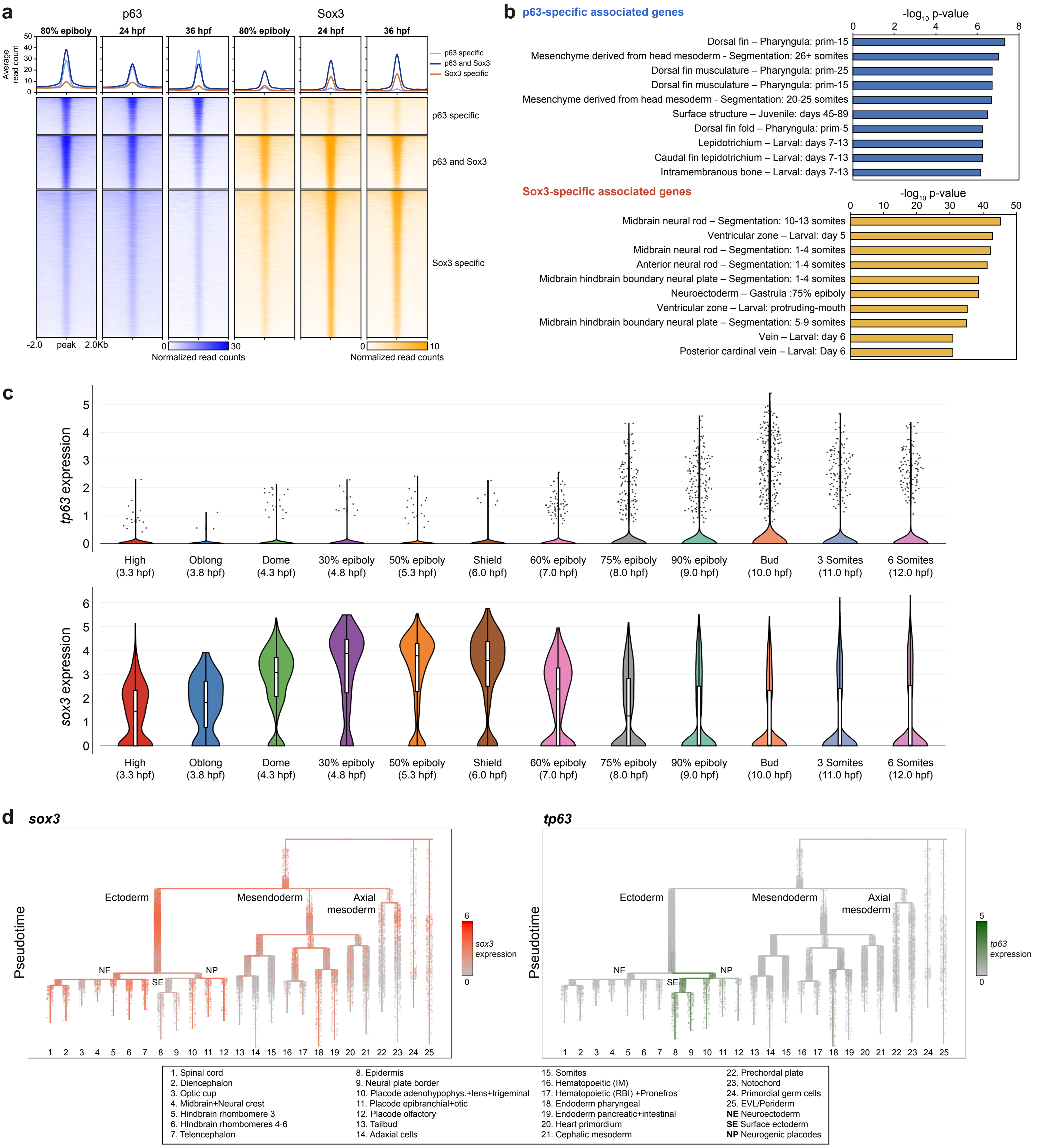

Supplementary Figure 2

Analysis of p63 and Sox3 binding to chromatin and the expression of their encoding genes during embryo development.

a, Heatmaps and average profiles of p63 and Sox3 binding in 80% of epiboly, 24 hpf and 36 hpf for the p63-specific, common and Sox3-specific peaks from Figure 3a. **b,** Top-10 enrichment of WT expression patterns of the genes corresponding to the p63- (top) and Sox3-specific (bottom) peaks from Figure 3a. The -$\log_{10}$ of p-value for each term is shown. **c,** Violin plots showing *tp63* and *sox3* expression through early development in zebrafish single-cell RNA-seq data[46]. **d,** *sox3* and *tp63* expression across developmental trajectories of zebrafish embryos. Tree representation based in lineage tracing of zebrafish early development by single cell RNA-seq[46], showing the expression of *sox3* in red (left) and *tp63* in green (right).

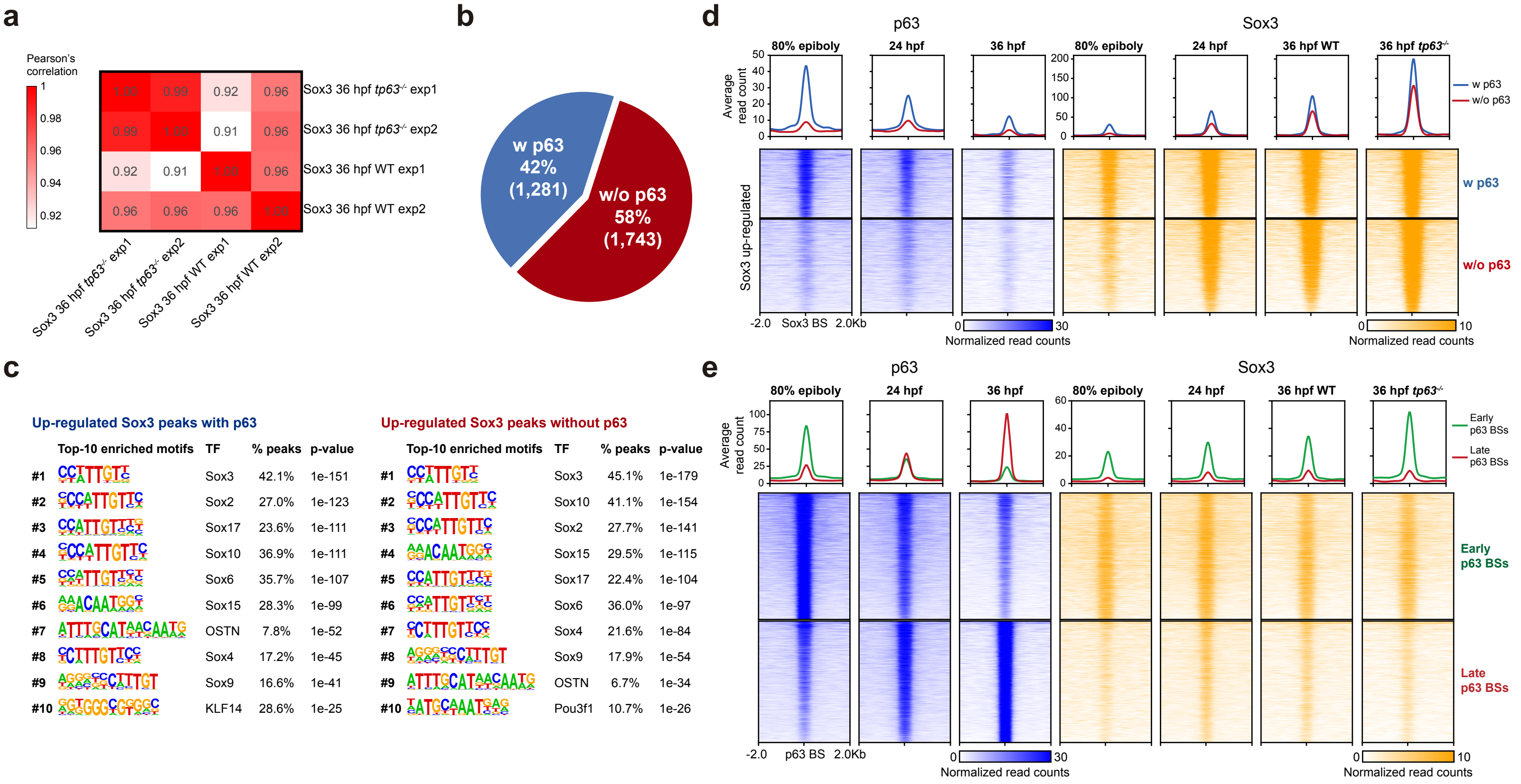

Supplementary Figure 3

Analysis of Sox3 binding to chromatin in *tp63*$^{-/-}$ mutants.

a, Heatmap showing Pearson's correlations between Sox3 ChIP-seq experiments in 36 hpf stage. **b,** Diagram showing the proportion of the 2,976 up-regulated Sox3 BSs in *tp63*$^{-/-}$ mutant from Figure 4a overlapping or not with p63 BSs. **c,** Motif enrichment analysis of the up-regulated Sox3 BSs overlapping or not with p63 BSs. The top-10 motifs are represented with their position in the top-10, motif logos, TF names, percentage of peaks with the motif and enrichment p-value. **d,** Heatmaps and average profiles of p63 and Sox3 binding in 80% of epiboly, 24 hpf and 36 hpf for the up-regulated Sox3 BSs overlapping or not with p63 BSs. Sox3 data for *tp63*$^{-/-}$ mutant at 36 hpf is also shown. For *b* and *d*, w, with; w/o, without. **e,** Heatmaps and average profiles of p63 and Sox3 binding in 80% of epiboly, 24 hpf and 36 hpf for the early and late p63 BSs from Figure 2b. Sox3 data for *tp63*$^{-/-}$ mutant at 36 hpf is also shown.

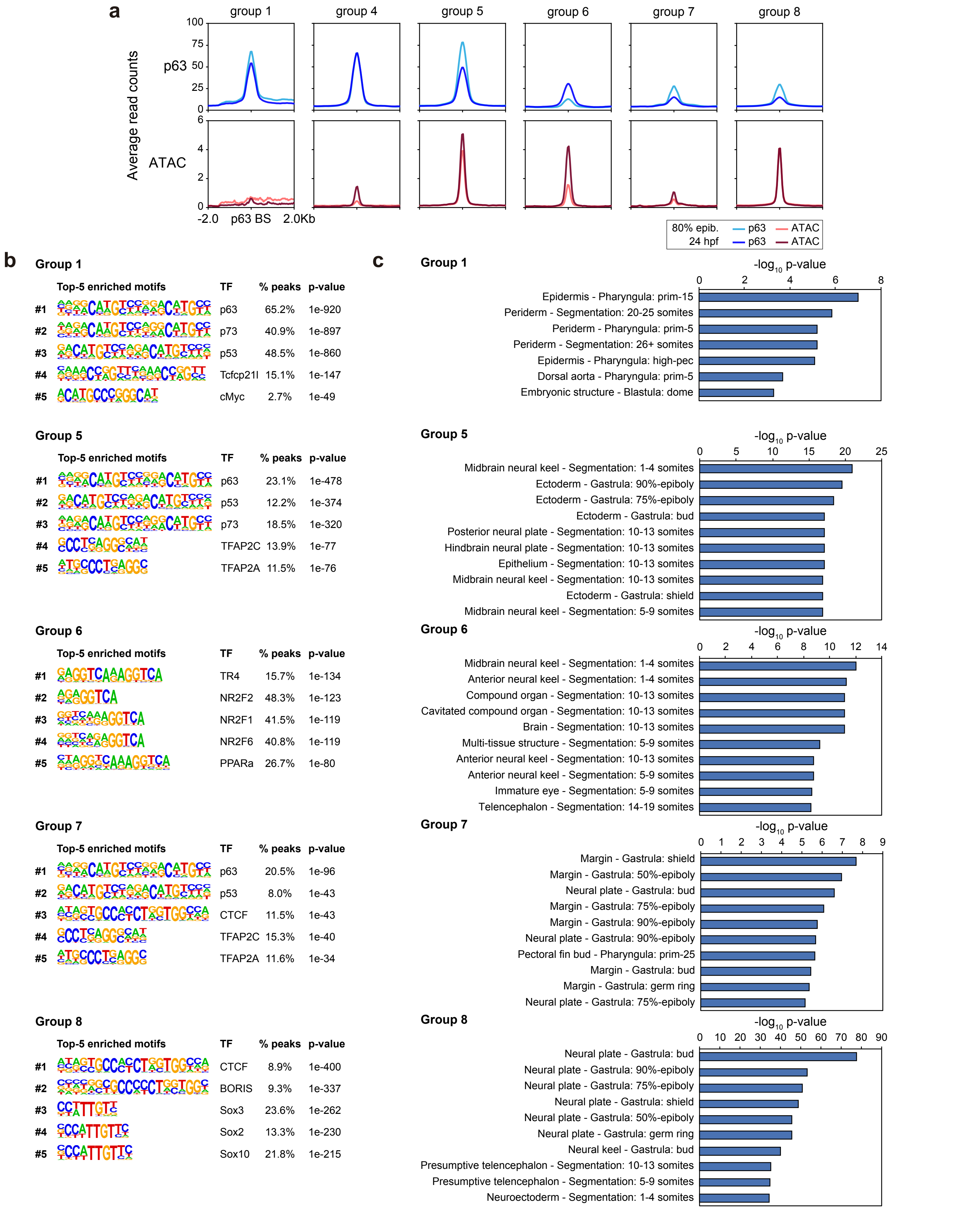

Supplementary Figure 4

Analysis of p63 binding and chromatin accessibility during embryo development.

a, Average profiles of p63 ChIP-seq and ATAC-seq from the peaks corresponding to transitions in the alluvial plot (see Figure 5a) between 80% of epiboly and 24 hpf stages. **b,** Motif enrichment analysis of the groups of peaks from alluvial plot (see Figure 5a). The top-5 motifs are represented with their position in the top-5, motif logos, TF names, percentage of peaks with the motif and enrichment p-value. **c,** Top-10 enrichment of WT expression patterns for the genes corresponding to the groups of peaks from alluvial plot (see Figure 5a). The -$\log_{10}$ of p-value for each term is shown. For *b* and *c*, data for group 4 are included in Figure 5d and e, respectively.

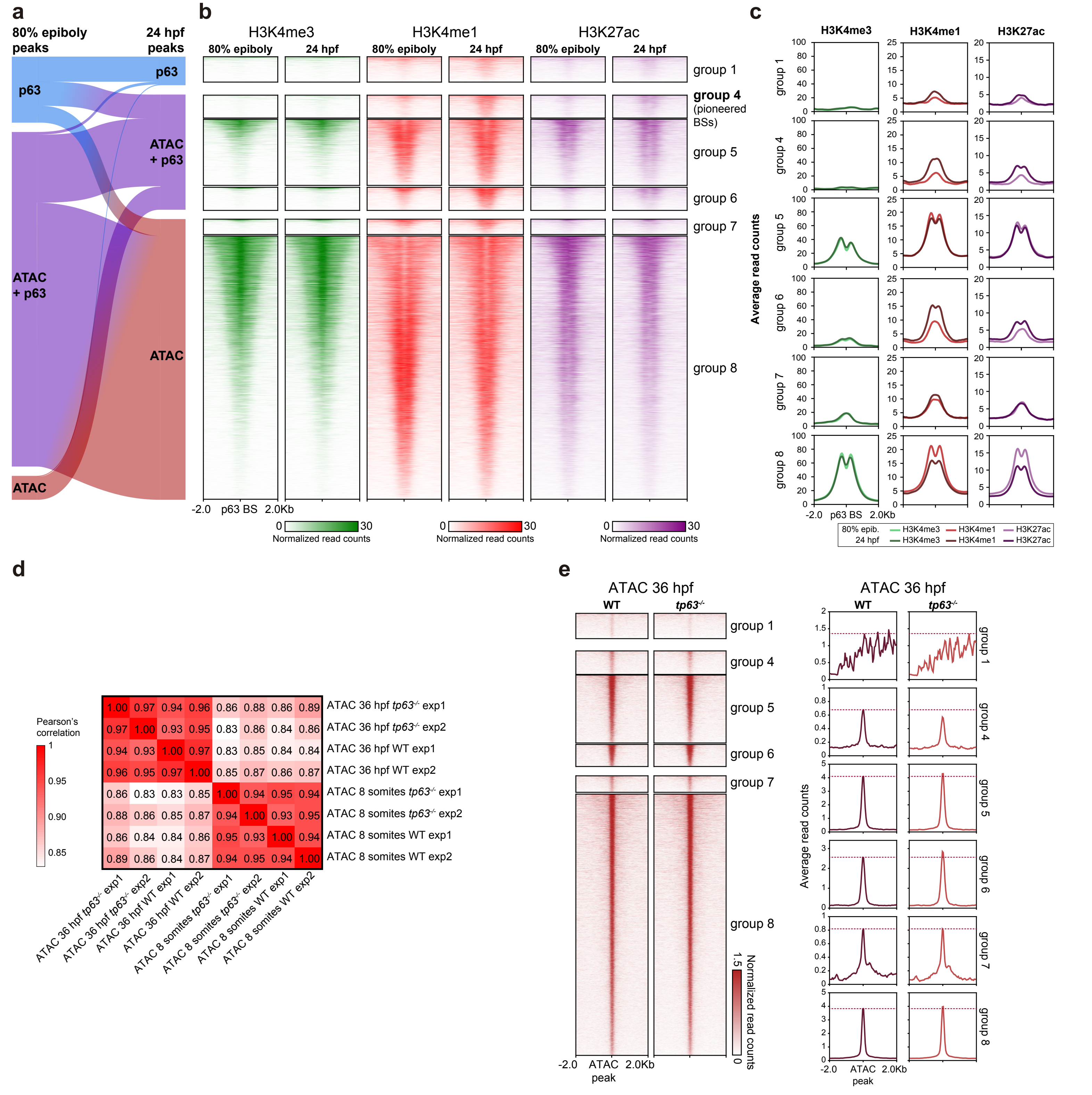

Supplementary Figure 5.

Epigenomic and p63-dependent chromatin accessibility analyses of p63 BSs.

a, Alluvial plot showing the transition between 80% of epiboly and 24 hpf stages of the 22,583 peaks showing p63 binding (blue), chromatin accessibility (ATAC; red) or both (purple). See also Figure 5. **b,** Heatmaps of H3K4me3, H3K4me1 and H3K27ac signal in 80% of epiboly and 24 hpf stages for the groups resulting from the alluvial plot transitions in *a*. **c,** Average profiles of H3K4me3, H3K4me1 and H3K27ac signal intensities for the groups resulting from the alluvial plot transitions in *a*. **d,** Heatmap showing Pearson's correlations between ATAC-seq experiments in 8 somites and 36 hpf stages. **e,** Heatmaps and average profiles of ATAC-seq from WT and *tp63*$^{-/-}$ embryos in 36 hpf for the groups resulting from the alluvial plot transitions in *a*. A dotted red line represents the WT profile peak in each stage. Groups 2 and 3 were omitted from the analyses due to their small number of peaks.

Supplementary Table 1 – List of primers used in this study

Primer name	Primer sequence
sgRNA_tp63_exon3	5′-TAATACGACTCACTATAGGCCGTATGACTGCACCC TGGTTTTAGAGCTAGAA-3′
sgRNA_universal	5′-AAAAGCACCGACTCGGTGCCACTTTTTCAAGTTGATAA CGGACTAGCCTTATTTTAACTTGCTATTTCTAGCTCTAAAA C-3′
tp63_ex3_Fw	5′-TCCACAGAGTTGAAGAAGCTG-3′
tp63_ex3_Rv	5′-ATCCTCCACGTATTGAGCGT-3′
