# Supplementary material for: Pioneer and repressive functions of p63 during zebrafish embryonic ectoderm specification
Source: Nat Commun. 2019 Jul 11;10:3049. doi: 10.1038/s41467-019-11121-z (PMC6624255; doi:10.1038/s41467-019-11121-z)
Supplement: Supplementary file 3 — Description of Additional Supplementary Files [file 41467_2019_11121_MOESM3_ESM.pdf]

Description of Additional Supplementary Files

File name: Supplementary Code 1

Description: Python script to calculate enrichment of gene expression patterns in wild-type zebrafish embryos.
