# Supplementary material for: Pioneer and repressive functions of p63 during zebrafish embryonic ectoderm specification
Source: Nat Commun. 2019 Jul 11;10:3049. doi: 10.1038/s41467-019-11121-z (PMC6624255; doi:10.1038/s41467-019-11121-z)
Supplement: Supplementary file 5 — Reporting Summary [file 41467_2019_11121_MOESM5_ESM.pdf]

nature research

Corresponding author(s): Juan J. Tena

Last updated by author(s): Jun 6, 2019

Reporting Summary

Nature Research wishes to improve the reproducibility of the work that we publish. This form provides structure for consistency and transparency in reporting. For further information on Nature Research policies, see Authors & Referees and the Editorial Policy Checklist.

Statistics

For all statistical analyses, confirm that the following items are present in the figure legend, table legend, main text, or Methods section.

n/a	Confirmed	
☐	☒	The exact sample size (*n*) for each experimental group/condition, given as a discrete number and unit of measurement
☐	☒	A statement on whether measurements were taken from distinct samples or whether the same sample was measured repeatedly
☐	☒	The statistical test(s) used AND whether they are one- or two-sided<br>*Only common tests should be described solely by name; describe more complex techniques in the Methods section.*
☒	☐	A description of all covariates tested
☐	☒	A description of any assumptions or corrections, such as tests of normality and adjustment for multiple comparisons
☐	☒	A full description of the statistical parameters including central tendency (e.g. means) or other basic estimates (e.g. regression coefficient) AND variation (e.g. standard deviation) or associated estimates of uncertainty (e.g. confidence intervals)
☒	☐	For null hypothesis testing, the test statistic (e.g. *F*, *t*, *r*) with confidence intervals, effect sizes, degrees of freedom and *P* value noted<br>*Give P values as exact values whenever suitable.*
☒	☐	For Bayesian analysis, information on the choice of priors and Markov chain Monte Carlo settings
☒	☐	For hierarchical and complex designs, identification of the appropriate level for tests and full reporting of outcomes
☒	☐	Estimates of effect sizes (e.g. Cohen's *d*, Pearson's *r*), indicating how they were calculated

Our web collection on statistics for biologists contains articles on many of the points above.

Software and code

Policy information about availability of computer code

Data collection	All code used to collect the data in this study is available in the Methods section
Data analysis	All code used to analyse the data in this study is available in the Methods section

For manuscripts utilizing custom algorithms or software that are central to the research but not yet described in published literature, software must be made available to editors/reviewers. We strongly encourage code deposition in a community repository (e.g. GitHub). See the Nature Research guidelines for submitting code & software for further information.

Data

Policy information about availability of data

All manuscripts must include a data availability statement. This statement should provide the following information, where applicable:

- Accession codes, unique identifiers, or web links for publicly available datasets
- A list of figures that have associated raw data
- A description of any restrictions on data availability

Sequencing data are available through the Gene Expression Omnibus accession number GSE123059

Field-specific reporting

Please select the one below that is the best fit for your research. If you are not sure, read the appropriate sections before making your selection.

☒ Life sciences ☐ Behavioural & social sciences ☐ Ecological, evolutionary & environmental sciences

For a reference copy of the document with all sections, see nature.com/documents/nr-reporting-summary-flat.pdf

Life sciences study design

All studies must disclose on these points even when the disclosure is negative.

Sample size	The number of embryos used for sample preparation was determined following previously reported methods, and allowed the extraction of enough biological material to perform the experiment. The number of replicates for each experiment was determined following the standards previously published for the ENCODE project. Sample sizes and number of replicates are specified in the methods section.
Data exclusions	For correlation between reads in peaks of ChIP and ATAC experiments, peaks with abnormally high number of reads were considered outliers and excluded. Two ChIP-seq experiments (second replicates of Sox3 in 80% epiboly and 24hpf) were excluded due to the low quality of the samples.
Replication	Biological replicates used in the study showed a high correlation, as specified in the supplementary material.
Randomization	Randomization of the samples is not applicable to our study, since no treatment conditions were compared. All comparisons were performed between different embryonic stages or between different genotypes, which do not require randomization.
Blinding	Blinding was not relevant for our study, since all comparisons were performed automatically using statistical software that is not influenced by the investigator.

Reporting for specific materials, systems and methods

We require information from authors about some types of materials, experimental systems and methods used in many studies. Here, indicate whether each material, system or method listed is relevant to your study. If you are not sure if a list item applies to your research, read the appropriate section before selecting a response.

Materials & experimental systems

n/a	Involved in the study	
☐	☒	Antibodies
☒	☐	Eukaryotic cell lines
☒	☐	Palaeontology
☐	☒	Animals and other organisms
☒	☐	Human research participants
☒	☐	Clinical data

Methods

n/a	Involved in the study	
☐	☒	ChIP-seq
☒	☐	Flow cytometry
☒	☐	MRI-based neuroimaging

Antibodies

Antibodies used	All antibodies used in the study are described in the Methods section.
Validation	All antibodies were validated by previous studies and/or the manufacturer. We also performed immunostaining assays as a validation.

Animals and other organisms

Policy information about studies involving animals; ARRIVE guidelines recommended for reporting animal research

Laboratory animals	This study did not require the use of adult animals. We used zebrafish embryos at different developmental stages derived from AB and Tübingen strains.
Wild animals	The study did not involve wild animals.
Field-collected samples	The study did not involve field-collected samples.
Ethics oversight	All experiments involving animals conform national and European Community standards for the use of animals in experimentation and were approved by the Ethical Committees from the University Pablo de Olavide, CSIC and the Andalusian government.

Note that full information on the approval of the study protocol must also be provided in the manuscript.

ChIP-seq

Data deposition

☒ Confirm that both raw and final processed data have been deposited in a public database such as GEO.

☒ Confirm that you have deposited or provided access to graph files (e.g. BED files) for the called peaks.

Data access links<br>*May remain private before publication.*	GEO datasets series: GSE123059
Files in database submission	For each sample, we submitted the fastq file with raw reads and the bed file with called peaks.
Genome browser session<br>(e.g. UCSC)	http://genome.ucsc.edu/cgi-bin/hgTracks?<br>hgS_doOtherUser=submit&hgS_otherUserName=jmsantos&hgS_otherUserSessionName=p63_NatCommun

Methodology

Replicates	No experimental replicates were performed. Instead, two biological replicates with high correlation among them were used per experiment.
Sequencing depth	p63_80epib_rep1: 36923847 total reads; 17876065 uniquely mapped reads; 49-bp paired-end reads<br>p63_80epib_rep2: 43003954 total reads; 20591645 uniquely mapped reads; 49-bp paired-end reads<br>p63_24hpf_rep1: 18409427 total reads; 7916396 uniquely mapped reads; 49-bp paired-end reads<br>p63_24hpf_rep2: 19289417 total reads; 9735783 uniquely mapped reads; 49-bp paired-end reads<br>p63_36hpf_rep1: 36567169 total reads; 4473514 uniquely mapped reads; 49-bp paired-end reads<br>p63_36hpf_rep2: 43364481 total reads; 742636 uniquely mapped reads; 49-bp paired-end reads<br>Sox3_80epib: 10007068 total reads; 3255385 uniquely mapped reads; 49-bp paired-end reads<br>Sox3_24hpf: 14743535 total reads; 7633687 uniquely mapped reads; 49-bp paired-end reads<br>Sox3_36hpf_WT_rep1: 42564819 total reads; 20571662 uniquely mapped reads; 49-bp paired-end reads<br>Sox3_36hpf_WT_rep2: 40441058 total reads; 20281402 uniquely mapped reads; 49-bp paired-end reads<br>Sox3_36hpf_tp63-/-_rep1: 45835872 total reads; 23596720 uniquely mapped reads; 49-bp paired-end reads<br>Sox3_36hpf_tp63-/-_rep2: 34252274 total reads; 17146347 uniquely mapped reads; 49-bp paired-end reads
Antibodies	All antibodies used in the study are described in the Methods section.
Peak calling parameters	macs2 callpeak -t experiment.bed -f BED -g 1.5e9 -n experiment-name -q 1e-3
Data quality	Peaks were called using a stringent cutoff (FDR<0.001; fold-change>5) and those common to both biological replicates were used as high-confidence peaks. The number of peaks for each experiment and replicate was:<br>p63_80epib_rep1: 36652 peaks FDR<0.001<br>p63_80epib_rep2: 42668 peaks FDR<0.001<br>p63_24hpf_rep1: 14707 peaks FDR<0.001<br>p63_24hpf_rep2: 25659 peaks FDR<0.001<br>p63_36hpf_rep1:16993 peaks FDR<0.001<br>p63_36hpf_rep2: 9538 peaks FDR<0.001<br>Sox3_80epib: 6045 peaks FDR<0.001<br>Sox3_24hpf: 67886 peaks FDR<0.001<br>Sox3_36hpf_WT_rep1: 51504 peaks FDR<0.001<br>Sox3_36hpf_WT_rep2: 29082 peaks FDR<0.001<br>Sox3_36hpf_tp63-/-_rep1: 81578 peaks FDR<0.001<br>Sox3_36hpf_tp63-/-_rep2: 59786 peaks FDR<0.001
Software	All the software used to collect and analyze the ChIP-seq data is described in the Methods section.
